# Effect of Amphetamine Dependence on Redox State via Alteration of Urinary Levels of Major and Trace Elements: A Case-Control Study in the Saudi Population

**DOI:** 10.7759/cureus.59819

**Published:** 2024-05-07

**Authors:** Lira A Al-Saif, Said A Aljawhri, Osama G Eissa, Rania H Mahmoud, Eman S Said

**Affiliations:** 1 Department of Pharmacology and Toxicology, College of Pharmacy, Qassim University, Buraydah, SAU; 2 Department of Laboratory, Erada Medical Rehabilitation Centre in Qassim, Ministry of Health, Buraydah, SAU; 3 Department of Psychiatry, Erada Medical Rehabilitation Centre in Qassim, Ministry of Health, Buraydah, SAU; 4 Department of Medical Biochemistry and Molecular Biology, Faculty of Medicine, Fayoum University, Fayoum, EGY; 5 Department of Medical Pharmacology, Faculty of Medicine, Fayoum University, Fayoum, EGY

**Keywords:** substance use disorder sud, redox status, heavy metals, trace elements, major elements, amphetamine

## Abstract

Background

Drug and substance abuse remains a major medical problem worldwide. Amphetamines are potent stimulants of the central nervous system. Amphetamine abuse is highly prevalent among drug-dependents. This study was conducted in Qassim, Saudi Arabia, to evaluate amphetamine's toxic effects on major and trace elements and their correlation with redox status.

Methods

The study involved amphetamine-only patients admitted to the Erada Rehabilitation Centre from March to October 2023. Urine samples were analysed from both normal subjects and amphetamine-dependent groups.

Results

Urinary sodium and chloride levels were significantly higher in the amphetamine-dependent group than in the control group, while their calcium levels decreased. Lipid peroxidase levels significantly increased in people with a substance use disorder (SUD), indicating oxidative stress. Together, their total antioxidant capacity decreased. Zinc (Zn), copper (Cu), lead (Pb), cadmium (Cd), sodium (Na), and total antioxidant capacity levels were positively correlated with lipid peroxidase.

Conclusions

Amphetamine-dependent people are more likely to experience a variety of health problems. This study found a direct correlation between an imbalance in major and trace elements and the redox status.

## Introduction

Drug and substance abuse remains a major medical problem worldwide [1]. Amphetamines are potent stimulants of the central nervous system. Amphetamine abuse is highly prevalent among drug-dependent people [2]. In 2015, the estimated global prevalence among the adult population was 18.4% for daily heavy alcohol use. Regarding tobacco smoking, the prevalence was 15.2% and 3.8% for cannabis. Opioid prevalence is 0.37% and 0.35% for cocaine [3]. The worldwide prevalence of amphetamine use among adults was 0.77% [3]. In Saudi Arabia, a retrospective study by the Psychiatric Rehabilitation Centre (PRC) of Buraydah identified amphetamine, alcohol, and cannabis as the most often abused drugs. Approximately 75% of substance use disorders (SUDs) fall within the 20-40 age bracket. Regarding the education of drug-dependent individuals, 66% did not complete high school, 16.5% did not finish elementary school, and 9.6% were college graduates [4,5].

Oxidative stress is the disparity between generating reactive oxygen species (ROS) and a biological system's capacity to eliminate these reactive substances [6]. Free radicals originate from internal and external sources. Endogenous free radicals are produced due to inflammation, infection, ischemia, cancer, mental stress, and aging. Certain medications, like cyclosporine, gentamycin, and bleomycin, can lead to the formation of exogenous free radicals. Consuming smoked meat, inhaling cigarette smoke, drinking alcohol, and radiation exposure all lead to an increase in free radical generation [7]. Elevated amounts of trace elements such as copper (Cu), zinc (Zn), cobalt (Co), manganese (Mn), and molybdenum (Mo) can lead to oxidative stress. Deficiency or excess trace elements can induce body metabolic disorders and cellular growth disturbance, including mutation and cancer development [8]. Malignant tumor development and progression are linked to elevated levels of trace elements such as selenium (Se), iron (Fe), and manganese (Mn) [8]. Trace elements, including cadmium (Cd), chromium (Cr), lead (Pb), and mercury (Hg), are crucial to biological structures and are toxic at high concentrations [8]. Excessive trace elements affect protein activity, modify gene expression, and thus disturb cellular functions, raising the likelihood of certain disorders [9].

A previous study conducted by Alharbi et al. in the Qassim region of KSA evaluated the health status outcome in amphetamine-dependents and demonstrated that oxidative stress biomarkers remained elevated. Although an improvement was observed in most of the parameters after the detoxification phase, oxidative stress biomarkers did not improve, which represents perminant harm to amphetamine users [5]. In this research, we aimed to correlate major and trace element levels and redox status among amphetamine-dependents.

## Materials and methods

Subjects

This study was conducted at the Erada Hospital for Mental Health Rehabilitation Centre, Qassim, KSA. It included 52 participants. Thirty-two were amphetamine-dependent (a test group), and 20 were healthy volunteers (a control group).

All patients were examined, and their complete medical history was recorded. We collected urine samples at the time of admission.

Study design, location, and duration

The present study used a comparative case-control study. The study was performed in a PRC at Erada Hospital in the Qassim region of Saudi Arabia. The study took place in the period between March and October 2023.

Sample selection

The control group was matched to the exposed group regarding lifestyle, age, and sex. All participants in this study were male. Medical history, smoking habits, and history of substance abuse disorder (including duration of substance use disorder, age of the participant, work environment, marital status, education level, and type of substance used) were obtained.

Inclusion and exclusion criteria

Table 1 lists the inclusion and exclusion criteria of this study.

**Table 1:** Inclusion and exclusion criteria.

Inclusion criteria	Exclusion criteria
Patients with positive amphetamine abuse.	Non-amphetamine-dependent.
Patients are more than 20 years old and less than 60 years old.	Patients who abuse amphetamines with other drugs.
Amphetamine abusers at concentrations equal to/more than 2000 ng/mL.	Any acute or chronic condition would limit the patient's ability to participate in the study.
	Refusal to give informed consent.
	Amphetamine abusers at concentrations less than 2000 ng/mL.

Ethics statement

The study received permission from the College of Pharmacy. The University Ethics and the Research Ethics Committee of the Ministry of Health in Saudi Arabia approved this research, with ethical approval number 607-44-013675. In addition, each participant (or their guardians) provided written informed consent after the purpose of the study was explained to them.

Study approach

Data

Information from each participant was collected using a pre-designed, three-part questionnaire. The first part contained questions about personal and socio-demographic data such as age, marital status, and unique lifestyle habits such as smoking and friends. The second part included questions relating to substance use disorder history, such as the reason behind substance dependence, motive for intake, duration of substance use disorder, and type of substance use disorder. The third part inquired about any past medical problems.

Study group

All participants in this study were free of any medical disease except for amphetamine abuse disorder and had similar dietary habits. The participants were then divided into two groups: group I, a control group (n = 20) of healthy persons, and group II, amphetamine-dependent (n = 32).

Clinical examination

The patient's history, including age, sex, unique habits, occupation, toxicological history, and medical history, was recorded, and a complete physical examination was performed.

Urine collection and storage

The urine voided was collected in a tightly capped polypropylene container and refrigerated immediately after urination until processed soon; otherwise, it was kept at −20 °C until analysis if there was a delay of more than 48 hours.

Determination of Urinary Amphetamine Level

Amphetamine levels in urine samples were measured using wet chemistry by the Architect c4000 + auto-analyzer (Abbott Co., North Chicago, IL) using reagents, methods, and approved lab policies. In QMHH, in addition to following manufacturer instructions, the assay principle utilizes the kinetic interaction of microparticle technology in solution. The amphetamine assay is a two-reagent system consisting of a microparticle-bound antibody with a drug-polymer conjugate in solution as the second reagent. Amphetamine urine assays of people with substance use disorders consider a result concentration of 2000 ng/mL or above as a lab. The clue for amphetamine DOA (range 0-300 ng/mL) is non-DOA towards amphetamine.

Determination of Urinary Total Antioxidant Capacity

Total antioxidant capacity was measured using the colorimetric technique.

Principle: The antioxidative capacity is assessed by reacting the antioxidants in the sample with a specific quantity of externally supplied hydrogen peroxide (H_2_O_2_). The antioxidants in the sample reduce a particular amount of the hydrogen peroxide provided. The remaining H_2_O_2_ is measured using calorimetry through an enzymatic reaction that converts 3,5-dichloro-2-hydroxybenzenesulfonates into a coloured product. The results were expressed as mmol/L of urine [10].

Determination of Urinary Lipid Peroxide

Urinary lipid peroxide was measured calorimetrically.

Principle: Thiobarbituric acid (TBA) reacts with malondialdehyde (MDA) in the acidic medium at 95 °C for 30 minutes to form a thiobarbituric acid-reactive product. The absorbance of the resultant pink product can be measured at 534 nm. The results were expressed as nmol/ml of urine [11].

Determination of Urinary Level of Major and Trace Elements

The urinary major (Na, Cl, K, Ca) and trace (Zn, Cu, Hg, Pb, and Cd) elements were measured using ICP-OES, or Inductively Coupled Plasma Optical Emission Spectroscopy (Optima® 8x00 ICP-OES) [12].

The ICP-OES principle: An analytical method called ICP-OES counts specific components in a sample. The ICP-OES principle transfers electrons from the ground state to an excited state by using the fact that atoms and ions may absorb energy. That energy is heat from an argon plasma running at 10,000 kelvins in ICP-OES [13].

Statistical analysis

The study used SPSS version 27 (IBM Corp., Armonk, NY) to analyse the data, with categorical data presented as numbers and percentages and numerical data tested for normality using the Shapiro-Wilk test. Chi-square or Fisher exact tests were used to test associations between variables. Independent T-tests were used to compare numerical data between groups, and Pearson and Spearman's rank correlations were used to investigate relationships between age and amphetamine intake with urine biomarkers.

## Results

Socio-demographic characteristics

The ages of the participants ranged from 21 to 56 years old. The mean ± SD were 34.4 ± 7.0 and 35.5 ± 10.9 for people with amphetamine use disorder and healthy individuals, respectively. There was no significant difference between the two studied groups (P > 0.05). Most of the participants were from urban areas; for amphetamine and healthy groups, 87.5% and 70.0%, respectively. The present study showed that 59.5% of the patients were single. For the healthy group, 60% were married. This work demonstrated that 59.4% of the people with amphetamine dependence were living alone, while 75% of the control group lived with their family. Regarding participants' educational level, the highest percentage of high secondary school graduates (46.9%) were in the amphetamine group; meanwhile, 60.0% were highly educated in the healthy participants, and 59.4% of amphetamine-dependents were jobless. Most healthy volunteers (65%) were employed. The present study showed that 56.4% of people with an amphetamine use disorder were caffeine users, 6.3% were cigarette smokers, and 45% of normal control individuals were caffeine users (Table 2).

**Table 2:** Sociodemographic characteristics of the studied groups. *Significant at p < 0.05.

		Groups				Fisher-Freeman-Halton Exact and Fisher’s Exact tests, Pearson’s chi-square	
		Amphetamine-dependent (N=32)		Healthy volunteers (N=20)			
		N	%	N	%	X^2^	P-value
Age, years	Mean ± SD	34.4 ± 7.0		35.5 ± 10.9		t = −0.389	0.700
Age groups	20–30	6	18.8	7	35.0	4.484	0.204
	31–40	22	68.8	8	40.0		
	41–50	2	6.3	2	10.0		
	51–60	2	6.3	3	15.0		
Residence	Rural	4	12.5	6	30.0	2.336	0.156
	Urban	28	87.5	14	70.0		
Marital status	Single	19	59.4	6	30.0	4.365	0.095
	Married	11	34.4	12	60.0		
	Divorced	2	6.3	2	10.0		
Social status	Lives alone	19	59.4	4	20.0	9.401	0.005*
	Lives with family	10	31.3	15	75.0		
	Living with friends	3	9.4	1	5.0		
Education level	High education	4	12.5	12	60.0	13.895	<0.001*
	High secondary	15	46.9	6	30.0		
	Intermediate	13	40.6	2	10.0		
Occupation	Employee	12	37.5	13	65.0	11.346	0.002*
	Unemployed	19	59.4	3	15.0		
	Student	1	3.1	4	20.0		
Special habits	Caffeine	18	56.3	9	45.0	4.828	0.155
	Smoking	2	6.3	0	0.0		
	Smoking and caffeine	3	9.4	0	0.0%		
	No	9	28.1	11	55.0		

Most amphetamine-dependent people do not have a family history of substance use disorder, and more than half of them suffer from an amphetamine-induced psychotic disorder. As recorded by this work, 43.8%, 6.3%, and 6.3% of the amphetamine group were associated with psychosis, schizophrenia, and anxiety, respectively. Friends' influence represented 34.4% of causes. In addition, factors such as lifestyle stress, frustration, and relationship issues were crucial contributors to the development of substance use disorder. The factors accounted for 15.6%, 6.3%, and 6.3%, respectively. The patients in the study were administered amphetamine for a period ranging from 2 to 11 years, with an average length of 5.4 ± 2.3 years (Table 3).

**Table 3:** Characteristics and psychiatric associations of amphetamine use disorder (N=32).

		N=32	%
Family history of substance use disorder	No	27	84.4
	Yes	5	15.6
Associations of amphetamine use disorder	No	14	43.8
	Psychosis	14	43.8
	Schizophrenia	2	6.3
	Anxiety	2	6.3
Addictive motives	Influence of friends	11	34.4
	Lifestyle stress	5	15.6
	Frustration	2	6.3
	Relationship difficulties	2	6.3
	Others	12	37.5
Duration of amphetamine use disorder	Minimum-maximum	2.0–11.0	
	Mean ± SD	5.4 ± 2.3	
Duration of intake	2 to <3	4 (12.5%)	
	3 to <4	4 (12.5%)	
	4 to <5	2 (6.3%)	
	≥5	22 (68.8%)	

Impact of amphetamine on major and trace elements

Urinary sodium ion levels and chloride ion levels were significantly increased in the amphetamine group compared to the control group. The median urinary sodium level was 113.5 mmol/L in the amphetamine group, compared to 78.5 mmol/L in the control group. The median urinary chloride level was 215.5 mmol/L in the amphetamine group compared to 186.0 mmol/L in the control group (Table 4).

**Table 4:** Urinary levels of major elements (essential electrolytes). *Significant at p < 0.05, IQR: interquartile range.

		Groups		Mann-Whitney U test	
		Amphetamine-dependent (N=32)	Healthy volunteers (N=20)	Zmw	P-value
Sodium (mmol/L)	Minimum-maximum	76.0–168.0	56.0–122.0	−3.739	<0.001*
	Median	113.5	78.5		
	IQR	93.0–125.0	76.0–98.5		
	Mean rank	32.70	16.58		
Chloride (mmol/L)	Minimum-maximum	101.0–355.0	99.0–260.0	−2.955	0.003*
	Median	215.5	186.0		
	IQR	199.0–231.0	183.5–188.5		
	Mean rank	31.41	18.65		
Potassium (mmol/L)	Minimum-maximum	53.50–175.09	44.30–123.50	−0.301	0.763
	Median	83.70	74.15		
	IQR	73.86–84.80	73.89–93.72		
	Mean rank	27.0	25.70		
Calcium (mmol/L)	Minimum-maximum	1.76–4.25	2.70–7.40	5.569	<0.001*
	Median	2.12	5.70		
	IQR	1.94–3.15	5.30–6.75		
	Mean rank	17.25	41.30		

Statistical analysis revealed a significant difference in the level of all trace elements between groups. The means ± SD of urinary levels of Zn in the amphetamine and control groups were 135.3 ± 30.1 and 106.5 ± 18.9, respectively. In comparison, the means ± SD of Cu were 89.0-205.9 and 54.3-144.0 in the amphetamine-dependent and control groups, respectively. Means ± SD of Hg were 20.10 ± 4.15 versus 4.50 ± 0.77 in both groups. Pb means ± SD were 16.91 ± 3.99 and 11.71 ± 1.54 in the people with amphetamine use disorder and the control group, respectively. Cd levels were 1.54 ± 0.39 versus 0.83 ± 0.08 in both groups. All these trace elements significantly increased in people with an amphetamine use disorder compared to the healthy group (P < 0.001) (Table 5).

**Table 5:** Urinary trace elements and heavy metals in the studied groups. *Significant at p < 0.05.

		Groups		Independent T-test	
		Amphetamine-dependent (N=32)	Healthy volunteers (N=20)	t	P-value
Level of trace elements in urine					
Zinc (μg/L)	Minimum-maximum	98.0–219.0	66.0–140.0	4.258	<0.001*
	Mean ± SD	135.3 ± 30.1	106.5 ± 18.9		
Copper (μg/L)	Minimum-maximum	89.0–205.9	54.3–144.0	3.520	<0.001*
	Mean ± SD	120.8 ± 28.3	94.6 ± 22.2		
Level of heavy metals in urine					
Mercury (μg/L)	Minimum-maximum	15.30–30.90	2.90–5.90	20.695	<0.001*
	Mean ± SD	20.10 ± 4.15	4.50 ± 0.77		
Lead (μg/L)	Minimum-maximum	10.22–27.80	8.10–15.40	6.620	<0.001*
	Mean ± SD	16.91 ± 3.99	11.71 ± 1.54		
Cadmium (μg/L)	Minimum-maximum	0.77–2.70	0.62–0.99	10.106	<0.001*
	Mean ± SD	1.54 ± 0.39	0.83 ± 0.08		

Impact of amphetamine on oxidative stress markers

The urinary lipid peroxidase level, a marker for oxidative stress, increased in the amphetamine-dependent group compared with the control group. The means ± SD in the amphetamine and control groups were 30.8 ± 7.3 and 9.5 ± 2.0, respectively. Conversely, the levels of total antioxidant capacity in urine substantially decreased with amphetamine use disorder compared with control (0.782 ± 0.178) and (1.506 ± 0.168), respectively (Table 6).

**Table 6:** Total antioxidant capacity and lipid peroxidase levels. *Significant at p < 0.05.

		Groups		Independent T-test	
		Amphetamine-dependent (N=32)	Healthy volunteers (N=20)	t	P-value
Total antioxidant capacity (mmol/L)	Minimum-maximum	0.554–1.200	1.262–1.683	−14.579	<0.001*
	Mean ± SD	0.782±0.178	1.506 ± 0.168		
Lipid peroxidase (nmol/ml)	Minimum-maximum	13.0–46.0	5.0–12.0	16.606	<0.001*
	Mean ± SD	30.8±7.3	9.5 ± 2.0		

Correlation Between Oxidative Stress Biomarkers, Major and Trace Elements

The levels of Zn, Cu, Pb, Cd, and Na in amphetamine-dependent urine were positively correlated with the level of lipid peroxidase. A statistically significant negative correlation was observed between Ca and lipid peroxidase. At the same time, the total antioxidant capacity was significantly correlated with Cu, Hg, Pb, Cd, and Na levels and then positively associated with calcium levels (Table 7).

**Table 7:** Correlations between the oxidative stress biomarkers and the studied urine biomarkers. *Significant at p <0.05, r: correlation coefficient.

	Total antioxidant capacity (mM/L)	Lipid peroxidase (nmol/ml)
	r	r
Zinc (μg/L)	−0.318	0.436*
Copper (μg/L)	−0.367*	0.417*
Mercury (μg/L)	−0.372*	0.344
Lead (μg/L)	−0.470*	0.471*
Cadmium (μg/L)	−0.526*	0.506*
Sodium (mmol/L)	−0.430*	0.400*
Chloride (mmol/L)	−0.038	−0.025
Potassium (mmol/L)	0.136	−0.038
Calcium (mmol/L)	0.909*	−0.878*

Correlations Between the Age of Amphetamine-Dependent and the Duration of Amphetamine Use Disorder With the Measured Biomarkers

Lipid peroxidase and lead levels were positively correlated with the duration of amphetamine intake. Either TAC or Ca was negatively correlated with the duration of amphetamine use disorder (Table 8).

**Table 8:** Correlations between the age of amphetamine-dependent and duration of amphetamine use disorder in the measured biomarkers. *Significant at p < 0.05, r: correlation coefficient.

	Age, years	Duration of intake
	r	r
Sodium (mmol/L)	−0.012	0.129
Chloride (mmol/L)	−0.147	−0.085
Potassium (mmol/L)	−0.005	−0.057
Calcium (mmol/L)	−0.033	−0.751*
Total antioxidant capacity (mmol/L)	−0.012	−0.736*
Lipid peroxidase (nmol/ml)	0.048	0.788*
Zinc (μg/L)	0.200	0.282
Copper (μg/L)	−0.120	0.073
Mercury (μg/L)	0.059	0.253
Lead (μg/L)	0.124	0.359*
Cadmium (μg/L)	−0.062	0.155

## Discussion

In Saudi Arabia, substance use disorder is considered a significant problem. The evidence of its association with psychiatric disorders and occupational losses is well documented [14]. Approximately 8% of Saudis have admitted to using illicit narcotics at some stage [15]. In 2014, Sweileh et al. documented a rise in amphetamine and cannabis consumption in Saudi Arabia compared to the previous decade [16]. In this clinical study, we describe, for the first time, to the best of our knowledge, the correlation between major and trace element levels and redox status among amphetamine-dependent. The study was conducted at the Erada Psychiatric Rehabilitation Centre, KSA.

This study revealed that there is a lack of female participants. This constraint was also noted in prior research carried out in the Qassim region by Ibrahim et al., who proved that female substance users were not included in their study [4]. Also, Saud Alharbi et al. showed a lack of female participants in their work [5]. This goes hand in hand with another research study conducted in a tertiary care hospital in Riyadh, which showed that only 12% of participants were women [17]. Ridley and Coleman found that over 50% of the hospitalized patients in Albany, Western Australia, were male [18]. Saquib et al. identified the male gender as a significant risk factor for substance use disorder in the Kingdom of Saudi Arabia [15]. The current study reaffirms the distinctive conditions involved in assessing substance use disorder (SUD) among females, as noted in prior research.

The findings of this study revealed that amphetamine-related disorders affect the most afflicted individuals aged between 31 and 40 years. Similar to the study of Ibrahim et al., who stated that the amphetamine abusers in their study were between 20 and 40 years old [4], global research showed a rising prevalence of substance misuse among young individuals, with amphetamine and cannabis being the most often used drugs [15]. The research showed that most drug users were in urban areas (Buraydah, Al-Rass, Unayzah, and Albadyaea). These findings align with the results of Alharbi et al., which show that many drug users were in urban areas (Buraydah, Al-Rass, Unayzah, and Albukayriah) [5]. Conversely, Roche and McEntee showed that rural and isolated areas in Australia have higher reported methamphetamine use compared to urban areas [19].

Regarding marital status, this study stated that most of the amphetamine group is single. Most amphetamine users in this study had a secondary school education, with a minority having attained a university-level education. Ibrahim et al. conducted a study at the Qassim Rehabilitation Centre, and their results were consistent with the findings of this study [4]. In 2016, Al-Musa and Al-Montashri performed a survey in the Aseer region of Saudi Arabia, namely, in all secondary schools in Abha city. The poll found that 8.8% of pupils were abusing illicit drugs [20]. Most of the amphetamine users in this clinical study were unemployed (59.4%). This could indicate possible criminal behaviour to acquire funds for purchasing illegal drugs [21]. According to a previous study by Qassim, stress contributes to the use of illicit drugs [4]. Substance misuse may be linked to the need to avoid the reality of the situation, as most users were unemployed [22].

This study revealed high urinary sodium and chloride excretion in the amphetamine group, which may lead to low plasma sodium. These findings agreed with hyponatraemia triggered by synthetic phenethylamines, which is associated with hypervolemia. This may be explained by increased antidiuretic hormone (ADH) secretion and consequent kidney water reabsorption. Hyponatremia is a big problem as it may trigger changes in mental status and lead to coma, seizures, cerebral oedema, and brain death [23].

Our research indicates that there was a considerable increase in urinary zinc levels in the amphetamine group. A prior study corroborates the current research. The results indicate a significant excretion of zinc in the urine of amphetamine users. Increased levels of zinc in urine can hinder the identification of methamphetamine, cocaine, and opiates by the enzyme-multiple immunoassay technique [24]. The study investigated the administration of zinc, either directly as an adulterant or through self-administration. Thus, it interferes with enzyme-based drug misuse detection in urine. The data show that zinc is added to urine 5,000 times higher than the average total zinc content in random urine samples. Thus, it can provide inaccurate adverse outcomes. Self-administration of oral zinc did not produce random urine zinc amounts within the required range [24]. This present study demonstrated higher urinary levels of mercury, lead, and cadmium in amphetamine-dependent groups compared with the healthy control group. Therefore, screening for blood heavy metal concentration is helpful for amphetamine-addict patients, especially those with nonspecific symptoms [25]. Drug dependents' urine contains high concentrations of heavy metals due to their adulteration with illicit drugs. Substance misuse and adulteration are prevalent when various active chemicals are added to medications for pharmacological or commercial reasons [26].

Similar to our findings, a recent study monitored urinary trace elements/heavy metals in individuals with substance use disorders (cannabis, opium, and tramadol). They found that these elements significantly increased in drug-dependent individuals compared to healthy individuals [27].

The noteworthy observation in this study is the significant decrease in total antioxidant capacity in the amphetamine group compared to the control group. There is a negative correlation between total antioxidant capacity and the duration of amphetamine use disorder. Previous research revealed that stimulant-dependent patients had substantially lower antioxidant capacity than controls and were more susceptible to oxidative tissue damage [28]. The data of the present study showed an elevation in lipid peroxidase in amphetamine users; lipid peroxidase is a biomarker of oxidative stress. Moreover, there was a positive correlation between lipid peroxidase and the duration of amphetamine use disorder. The results were consistent with Solhi et al., which demonstrated that long-term methamphetamine use causes oxidative stress in the body and increases lipid peroxidation. The incident could lead to negative consequences from both short-term and long-term methamphetamine use, including decreased attention, impaired motor skills, and cognitive impairments [29]. Antioxidants should be included in treatment regimens for methamphetamine abusers who seek medical care from physicians [29,30]. Amphetamine-induced mitochondrial reactive oxygen specious generation may be a valuable model to investigate the hypothesis of altered brain energy metabolism associated with bipolar disorder and schizophrenia [30].

Limitations of the study

The small sample size in the study is because most of the inpatients in the Erada centre are not amphetamine-dependent.

## Conclusions

This study indicates that amphetamine-dependents are more likely to experience many health problems. The urinary levels of major elements, sodium and chloride, as well as trace elements, Zn, Cu, Pb, and Cd, were significantly increased in the amphetamine-dependent group. This elevation of trace elements leads to oxidative stress, disturbs cellular functions, and raises the possibility of many diseases. Lipid peroxidase levels were significantly increased in amphetamine-dependent subjects, indicating oxidative stress. Regarding the primary goal of our investigations, the levels of Zn, Cu, Pb, Cd, and Na in amphetamine-dependent subjects' urine were positively correlated with the level of lipid peroxidase. A statistically significant negative correlation was observed between Ca and lipid peroxidase. Simultaneously, the total antioxidant capacity was statistically negatively correlated with amphetamine's Cu, Hg, Pb, Cd, and Na levels. We recommend regular assessment of heavy metal levels and oxidative stress within treatment for amphetamine use disorder, and we also recommend further studies to confirm our findings.
